# Dietary fermented cassava peel improves laying performance, yolk fatty acid profile, and economic return in laying hens

**DOI:** 10.14202/vetworld.2026.1629-1641

**Published:** 2026-04-25

**Authors:** Jamila Mustabi, Nuraini Nuraini, Denny Rusmana, Athhar Manabi Diansyah, Herdis Herdis, Maman Surachman, I Wayan Angga Darmawan, Windu Negara, Muhammad Nur Aqil Jamal

**Affiliations:** 1 Department of Nutrition and Feed Technology, Faculty of Animal Science, Hasanuddin University, Indonesia; 2 Department of Nutrition and Feed Technology, Faculty of Animal Science, Universitas Andalas, Padang, Indonesia; 3 Department of Animal Nutrition and Feed Technology, Faculty of Animal Husbandry, Padjadjaran, Bandung, Indonesia; 4 Department of Animal Production, Faculty of Animal Science, Hasanuddin University, Indonesia; 5 Research Center for Animal Husbandry, National Research and Innovation Agency (BRIN), Bogor, Indonesia

**Keywords:** cassava peel fermentation, economic efficiency, egg production, feed conversion ratio, laying hens, *Lentinus edodes*, solid-state fermentation, yolk fatty acids

## Abstract

**Background and Aim:**

Cassava peel is an abundant agro-industrial byproduct with feed potential, but its high fiber and antinutritional content limit direct use. Fermentation with *Lentinus edodes* may improve its nutritional quality. This study evaluated graded levels of *L. edodes*–fermented cassava peel (FCP) in laying hen diets for effects on nutrient composition, performance, yolk lipid profile, and economic return.

**Materials and Methods:**

A 12-week completely randomized trial involved 200 laying hens assigned to four dietary treatments (0, 5, 10, and 15% *L. edodes*–FCP; five replicates of 10 hens each). Cassava peel was fermented with *L. edodes*, dried, and milled before diet formulation. Diets met nutrient requirements and were analyzed for proximate composition and amino acids. Performance parameters included egg production, egg weight, feed conversion ratio (FCR), and hen-day production (HDP). Yolk fatty acids were determined by gas chromatography with flame ionization detection following Folch extraction and classified as saturated fatty acids (SFA), monounsaturated fatty acids (MUFA), and omega-3, omega-6, and omega-9. Income over feed cost (IOFC) was calculated from feed intake, egg output, and market prices.

**Results:**

*L. edodes*–FCP modified diet composition dose-dependently, increasing crude fiber and ether extract while reducing crude protein and lysine; metabolizable energy peaked at 5%. Egg production and HDP were highest at 5%, whereas egg weight and FCR were unaffected. Yolk SFA and MUFA remained stable, but omega profiles shifted: omega-3 and omega-9 increased at 5% and 15%, while omega-6 peaked at 10% and was lowest at 15%. Higher yolk MUFA and lower SFA were associated with increased laying rate, and omega distribution aligned with dietary energy and sulfur amino acid levels. IOFC was maximized at 5% (≈172% of control) but declined at 10%–15% due to reduced output. Overall, 5% inclusion represents the optimal level for performance and profitability.

**Conclusion:**

*L. edodes*–FCP can be safely incorporated into laying hen diets, with 5% inclusion optimizing performance and profitability. At this level, egg production and HDP increased without affecting egg weight or FCR, and IOFC improved. Higher inclusion levels reduced laying rate, likely due to increased dietary fiber. Yolk lipid profile was favorably modified, particularly with increased omega-3 and omega-9 fatty acids.

## INTRODUCTION

Feed costs dominate the economics of table egg production, which motivates the search for locally available ingredients that stabilize ration prices while safeguarding performance and product quality [1]. In Southeast Asia, processing of cassava (*Manihot esculenta*) generates substantial peel by-products that are often underutilized, despite their carbohydrate-rich matrix, which is suitable for valorization [2]. However, cassava peel contains high structural fiber, lignin, and cyanogenic glycosides that can release hydrogen cyanide (HCN), posing potential toxicity risks in monogastric animals if inadequately processed. Detoxification strategies such as drying, soaking, and especially microbial fermentation have been shown to markedly reduce cyanide content while improving nutrient availability, thereby enabling safer dietary inclusion [3].

Solid-state fermentation represents a scalable approach to upgrading lignocellulosic residues by depolymerizing cell wall polysaccharides, reducing antinutritional factors, and increasing nutrient accessibility [4]. Among edible white-rot fungi, *Lentinus edodes* (shiitake mushroom) is a basidiomycete widely cultivated for food and recognized for its ligninolytic enzyme system, including cellulases, hemicellulases, and laccases, which selectively degrade lignin and structural carbohydrates under mild conditions [5]. Beyond fiber degradation, *L. edodes* fermentation may increase crude protein (CP) through microbial biomass enrichment and reshape phenolic profiles and generate bioactive metabolites that influence gut physiology and lipid metabolism [6], enhancing its potential as a functional feed modifier rather than a neutral diluent.

In Indonesia, where maize and soybean meal prices are highly volatile, the need for locally sourced, requirement-balanced alternatives is particularly acute [7]. Robust evaluation in laying hens requires accurate pen-level feed intake, daily egg recording, standardized egg weight measurements, and matched-interval feed conversion ratio (FCR) calculations to accurately capture efficiency responses [8]. Previous studies have demonstrated that *L. edodes* fermentation enhances the feeding value of lignocellulosic residues by improving nutrient accessibility and reducing structural fiber content, thereby supporting their application in animal nutrition. In laying hens, fermented plant ingredients can sustain intake and egg production when diets are properly balanced; however, outcomes are strongly influenced by substrate characteristics, fermentation parameters, and inclusion level. Consequently, ingredient-specific validation under practical layer production systems remains essential before large-scale adoption [9]. Previous studies indicate that fermented plant ingredients can maintain or enhance laying rate and feed efficiency in nutritionally balanced diets; however, responses remain substrate- and dose-dependent [8]. Accordingly, a focused evaluation of *L. edodes*–fermented cassava peel (FCP) in laying hens, with rigorous intake and FCR accounting, is directly relevant for de-risking farm-level adoption.

Interest has also shifted toward yolk lipid quality, as dietary strategies can modify the relative abundance of omega-3, omega-6, and omega-9 fatty acids. In addition to influencing product differentiation, enrichment of omega-3 fatty acids is associated with recognized consumer health benefits, including anti-inflammatory and cardioprotective effects, thereby supporting value-added egg positioning. Matrix modifications induced by fermentation, including cell wall loosening and altered viscosity, may influence digestion and micellization, thereby affecting hepatic lipid deposition even when total fatty acid supply is similar [10]. Fermented plant ingredients have been associated with changes in desaturase and elongase activity and selective partitioning among omega fatty acid classes, although the magnitude and direction vary with substrate and dosage [11]. Whether *L. edodes*–FCP beneficially, neutrally, or adversely modulates yolk fatty acid composition in laying hens remains unclear, warranting controlled evaluation.

From a practical perspective, any alternative ingredient must meet economic feasibility criteria. Income over feed cost (IOFC) integrates ration cost, intake, egg output, and prevailing prices into a single profitability indicator [12]. Because *L. edodes*–FCP may simultaneously influence diet cost, intake behavior, production rate, and egg quality, assessing IOFC across graded inclusion levels provides a direct bridge between experimental findings and farm-level decision-making [13]. However, comprehensive evaluations integrating performance, yolk omega distribution, and profitability under local price conditions remain limited in the current literature for laying hens [14].

Although several studies have explored the use of fermented agro-industrial by-products in poultry diets, comprehensive evaluations that simultaneously assess production performance, yolk fatty acid profile, and economic return under local tropical conditions remain limited, particularly for *L. edodes*–FCP. Most previous investigations have focused on single parameters, such as laying rate or FCR, often without rigorous pen-level feed intake recording or matched-interval calculations, resulting in an incomplete understanding of dose-dependent responses. Furthermore, while fermentation with white-rot fungi is known to reduce antinutritional factors and improve nutrient accessibility, the specific effects of graded levels of *L. edodes*–FCP on the distribution of yolk omega-3, omega-6, and omega-9 have not been systematically examined, despite growing consumer interest in omega-enriched eggs. Existing reports are also largely substrate- and strain-specific, with insufficient data on how moderate versus high inclusion levels influence dietary energy density, sulfur amino acid balance, and subsequent hepatic lipid partitioning in laying hens. Critically, few studies have integrated biological outcomes with IOFC under prevailing farm-gate prices in Southeast Asia, where volatile maize and soybean meal costs make locally sourced alternatives essential. This gap hinders the development of evidence-based recommendations for circular bioeconomy-oriented feed strategies and limits the practical adoption of FCP in commercial layer production systems.

The present study therefore aimed to evaluate the effects of graded dietary inclusion (0, 5, 10, and 15%) of *L. edodes*–FCP in nutritionally balanced laying hen diets on nutrient composition, production performance (egg production, egg weight, hen-day production), feed efficiency (feed intake and FCR), yolk fatty acid profile (saturated fatty acids [SFA], monounsaturated fatty acids [MUFA], and omega-3, omega-6, and omega-9 series), and economic return (IOFC) under local Indonesian conditions. By integrating these parameters within a single 12-week, completely randomized trial using pen-level replication, the study sought to identify the optimal inclusion level that maximizes both biological performance and profitability while elucidating dose-dependent shifts in yolk lipid quality. This integrated approach provides practical inclusion thresholds and contributes to sustainable utilization of cassava peel waste in tropical layer production systems.

## MATERIALS AND METHODS

### Ethical approval

All experimental procedures involving laying hens were reviewed and approved by the Ethics Committee, Faculty of Medicine, Universitas Andalas, Indonesia, under Approval No. 0376/UN.16.2/KEP-FK/2025. The study was conducted in accordance with institutional guidelines for the care and use of animals in research. Throughout the experiment, the birds were managed under standard husbandry conditions, with free access to feed and water, routine health observation, and daily welfare monitoring. Housing, sanitation, ventilation, temperature, and relative humidity were maintained within appropriate ranges to minimize stress and ensure bird welfare. No invasive surgical procedures were performed during the study. All handling, egg collection, and sampling procedures were carried out carefully to avoid unnecessary distress, pain, or injury to the animals. Humane endpoints were established in advance, and any bird showing signs of severe illness, pain, or distress would have been promptly removed and treated appropriately; however, no such cases were observed during the study. The experiment was therefore performed in compliance with accepted ethical principles for animal welfare and scientific research.

### Study period and location

This study was conducted from June to December 2025 at the Faculty of Animal Science, Universitas Hasanuddin, Makassar, Indonesia.

### Study design

A completely randomized design (CRD) was used with four dietary treatments based on *L. edodes*–FCP inclusion: 0, 5, 10, and 15% of the diet (T0–T3). Two hundred hens (Hy-Line Brown, 28 weeks of age at the start of the trial, obtained from a commercial layer farm in South Sulawesi, Indonesia) were randomly allocated into 5 replicate pens per treatment with 10 hens per pen (pen = experimental unit). The average initial body weight was 1.75 ± 0.08 kg, with flock uniformity of approximately 90% based on body-weight distribution. All hens were of the same age and physiological stage to minimize production variability among treatments.

The feeding period lasted 12 weeks after a 7-day acclimation period. During the 12-week experimental period, mortality was 0%, and no birds were culled due to health disorders. Daily health monitoring revealed no clinical signs of toxicity or adverse effects associated with dietary FCP inclusion, confirming its safety within the tested range. Hens were housed under standard management conditions at an ambient temperature of approximately 27–30°C with relative humidity of 65–75%, typical of tropical open-sided housing systems. Feed and water were provided ad libitum. Ventilation was maintained naturally, and housing hygiene was monitored daily to prevent excess moisture accumulation. No visible mold contamination was observed in feed or litter during the study period. Birds were visually inspected daily for general health status, feed consumption behavior, and any signs of abnormality or distress. No automated intake monitoring systems (e.g., RFID-based individual tracking) were used; measurements were conducted at the pen level, which served as the experimental unit.

### Fermentation of cassava peel and diet formulation

Fresh cassava peel (*Manihot esculenta*) were washed and air-dried prior to fermentation. A commercially available culture of *L. edodes* was used as the inoculum and was initially propagated on a standard growth medium before being applied to the cassava peel substrate under solid-state fermentation conditions. Fermentation was conducted for 10 days at 25°C–28°C with relative humidity maintained at approximately 70%–80% [15]. Substrate moisture was adjusted to support fungal growth while preventing excessive wetness that could promote undesirable microbial proliferation. The material was inspected regularly to monitor mycelial colonization and detect potential contamination, and no abnormal microbial growth was observed. Upon completion of fermentation, the substrate was oven-dried at 55°C–60°C to constant weight to terminate microbial activity, then milled into a fine meal and designated as *L. edodes*–FCP for dietary inclusion.

Four experimental diets were prepared with graded *L. edodes*–FCP levels of 0, 5, 10, and 15% (T0–T3), while the remaining ingredients of the basal ration were proportionally adjusted so that metabolizable energy and key nutrients met the requirements of laying hens. The ingredient formulation of the experimental diets is presented in Table 1.

**Table 1:** Ingredient formulation of experimental diets (% as-fed).

Ingredient	T0	T1	T2	T3
Ground corn	50.00	50.00	50.00	50.00
Rice bran	15.00	15.00	15.00	15.00
Concentrate	35.00	30.00	25.00	20.00
Fermented cassava peel	0.00	5.00	10.00	15.00

The concentrate used in the experimental diets consisted primarily of soybean meal, fish meal, limestone, dicalcium phosphate, salt, vitamin–mineral premix, and synthetic amino acids, formulated to complement the basal ingredients. All feed ingredients were obtained from local commercial suppliers in Makassar, Indonesia. Diets were formulated to meet or exceed the nutrient requirements for laying hens according to National Research Council (NRC) [16] recommendations. The experimental rations were prepared in batch form and mixed using a horizontal feed mixer to ensure homogeneity before distribution to the laying hens.

### Nutrient composition of the experimental diets

Representative samples of each experimental diet (T0, T1, T2, and T3) were collected during feed preparation. Approximately 100 g subsamples were taken from multiple points within each batch to ensure representativeness, pooled per treatment, homogenized, and ground to pass through a 1-mm sieve prior to chemical analysis. All analyses were performed in triplicate, and results were expressed on an as-fed basis to correspond with diet formulation.

Dry matter and CP were determined according to AOAC method 934.01 standard procedures [17]; CP was analyzed by the Kjeldahl method (AOAC 990.03; N × 6.25) and expressed as a percentage of the diet. Crude fat was measured by ether extraction using a Soxhlet apparatus (AOAC 920.39), and crude fiber (CF) was determined using the conventional Weende method (AOAC 978.10) [18], both expressed as % of the diet. Apparent metabolizable energy (ME) was estimated from ingredient composition using tabulated poultry ingredient values and standard prediction equations for compound feeds, based on NRC guidelines [16], and expressed as kcal/kg.

Amino acid composition (lysine, methionine, methionine + cystine, threonine, and tryptophan) was analyzed by a certified commercial laboratory using ion-exchange high-performance liquid chromatography following acid hydrolysis (6N HCl, 24 h, 110°C). Performic acid oxidation was applied for sulfur-containing amino acids, and alkaline hydrolysis was used for tryptophan determination, in accordance with AOAC [19] procedures. Amino acid quantification was performed using external calibration with authenticated amino acid standards.

### Production performance and feed efficiency

Production performance was monitored throughout the 12-week feeding period. Eggs were collected daily from each pen at approximately 08:00 h and recorded to determine daily egg production. Egg numbers per pen were compiled weekly and cumulatively throughout the trial. On designated weighing days (once weekly), all eggs produced per pen were individually weighed using a calibrated digital balance (accuracy ±0.01 g) to calculate mean egg weight (g) at the pen level [20].

Feed intake was determined by recording the amount of feed offered and the amount refused per pen. Feed was weighed weekly using a digital scale (accuracy ±0.1 g), and total feed intake was calculated as feed offered minus refusals. Egg mass (kg) was calculated as total egg number × average egg weight (kg) per pen over the same period. FCR was computed as total feed intake (kg) divided by total egg mass (kg) for each pen to ensure consistency between intake and production outputs [21].

Hen-day production (HDP, %) was calculated daily as (number of eggs laid per day/number of live hens in the pen) × 100 and averaged over the experimental period [22]. Birds were visually inspected daily for general health status, feed consumption behavior, and any signs of abnormality or distress. No automated intake monitoring systems (e.g., RFID-based individual tracking) were used; measurements were conducted at the pen level, which served as the experimental unit.

### Egg yolk fatty acid profile

Eggs were collected daily and stored at ≤4 °C until analysis. For fatty acid determination, eggs were sampled during the final week of the feeding period. From each replicate pen, five eggs were randomly selected and pooled to obtain one composite sample per replicate. Yolks were separated, homogenized for at least 30 s, and aliquoted prior to extraction [23].

Approximately 2 g of homogenized yolk was used for lipid extraction using the Folch method (chloroform: methanol, 2:1, v/v), with 0.01% BHT as an antioxidant. The organic phase was washed with 0.88% KCl, dried over anhydrous Na_2_SO_4_, and the solvent was evaporated under a stream of N_2_ at ≤35°C. Fatty acid methyl esters (FAMEs) were prepared according to AOAC 996.06/ISO 5509 using base-catalyzed transesterification in methanolic KOH followed by acid methylation with BF_3_–methanol. FAMEs were extracted into n-hexane, washed with saturated NaCl, dried over anhydrous Na_2_SO_4_, and adjusted to a known final volume. FAMEs were separated by gas chromatography with flame ionization detection (GC-FID) using a polar cyanopropyl column (e.g., SP-2560; 100 m × 0.25 mm × 0.20 μm). Injector and detector temperatures were set at 260°C and 270°C, respectively. Helium was used as the carrier gas at approximately 1.0 mL min^−1^ with a split ratio of 1:50 and an injection volume of 1 μL. The oven temperature program began at 140 °C (held for 5 min), increased at 4°C min^−1^ to 240°C, and was held for 15 min. Peaks were identified by comparison with authenticated FAME standards and quantified using methyl nonadecanoate (C19:0) as an internal standard. Each sample was injected in duplicate, and quality control included blank runs and periodic calibration using reference standards to verify retention time stability and peak resolution [24].

The egg yolk fatty acid profile was expressed both as individual fatty acids and as grouped classes: SFA, MUFA, and polyunsaturated fatty acids of the n-3, n-6, and n-9 series, expressed as a percentage of total identified FAME. Fatty acid data are additionally expressed as a percentage of the total identified FAME to facilitate comparison with published literature.

### Economic outcomes

Economic outcomes were determined by IOFC. Egg revenue was calculated from cumulative egg production per pen multiplied by the prevailing farm-gate price per egg at the study site. The unit cost of *L. edodes*–FCP was estimated based on the availability of local cassava peel and processing expenses (including inoculum, labor, drying, and milling). In general, FCP was lower in cost than conventional energy sources, such as maize, and reduced the overall cost of the formulated diets as its inclusion level increased. Consequently, higher dietary FCP levels were associated with progressive reductions in feed cost per kilogram of diet. Feed cost per pen was calculated as total feed intake (kg) × diet cost (IDR/kg). Egg revenue per pen was calculated as total egg number × egg price (IDR/egg). Feed expenditure was derived from total feed consumed per pen multiplied by the respective diet cost. IOFC was expressed both in absolute terms and relative to the control (T0 = 100%) to illustrate proportional economic changes across treatments [25].

### Statistical analyses

The study employed a completely randomized design (CRD) with the pen as the experimental unit. Statistical analyses were performed using SPSS version 27.0 (IBM Corp., Armonk, NY, USA). Treatment effects of dietary *L. edodes*–FCP inclusion (0, 5, 10, and 15%) on nutrient composition, production performance (egg production, egg weight, hen-day production), feed efficiency (FCR), and yolk fatty acid profile (SFA, MUFA, n-3, n-6, and n-9) were analyzed using one-way analysis of variance. Data are presented as means ± standard error of the mean (SEM). Normality and homogeneity of variance assumptions were verified using the Shapiro–Wilk and Levene’s tests, respectively. When the overall F-test was significant (α = 0.05), differences among treatment means were determined using Tukey’s honestly significant difference test and are indicated by compact letter displays. Pearson correlation coefficients (r) were calculated to assess associations between production variables and yolk fatty acid classes. IOFC was evaluated descriptively based on treatment-level economic returns.

## RESULTS

### Nutritional composition

As shown in Table 2, the inclusion of *L. edodes*–FCP significantly altered the energy and nutrient profiles of the diets (p < 0.05), except for threonine. ME was highest in the diet containing 5% FCP (T1) and was significantly greater (p < 0.05) than T0, T2, and T3, whereas the other treatments did not differ from each other. In contrast, CP decreased with FCP inclusion: the control diet (T0) had a higher CP concentration than all FCP-supplemented diets (T1–T3; p < 0.05), which did not differ from one another. Fiber and fat fractions increased at the higher inclusion levels. Both CF and crude fat were significantly greater (p < 0.05) in the 10% and 15% FCP diets (T2 and T3) compared with T0 and T1, while T0 and T1 did not differ from each other. For the amino acid profile, lysine was highest in the control diet and significantly reduced in all FCP-containing diets (p < 0.05). Methionine was slightly increased at 10% FCP (T2), which differed from the other treatments (p < 0.05), whereas methionine + cystine showed a progressive decline with increasing FCP, with T0 and T1 having the highest values, T2 intermediate, and T3 the lowest (p < 0.05). Threonine concentration differed slightly among treatments (p < 0.05), while tryptophan was reduced at 5% and 10% FCP (T1 and T2) relative to T0 and T3 (p < 0.05). Overall, these findings indicate that FCP inclusion modified dietary energy density, fiber content, and key amino acids, particularly lysine and sulfur amino acids.

**Table 2:** Nutritional composition of experimental diets.

Parameter	T0	T1	T2	T3	SEM	p-value
Metabolizable energy (ME; kcal/kg)	2606.82ᵃ	2689.80ᵇ	2505.07ᵃ	2658.99ᵃ	252.51	0.035
Crude protein (%)	15.52ᵇ	14.89ᵃ	14.83ᵃ	13.92ᵃ	1.76	0.004
Crude fiber (%)	4.91ᵃ	5.10ᵃ	5.59ᵇ	5.66ᵇ	0.75	0.000
Crude fat (%)	3.91ᵃ	4.15ᵃ	4.44ᵇ	4.36ᵇ	0.56	0.000
Lysine (%)	0.75ᵇ	0.73ᵃ	5.59ᵃ	0.69ᵇ	0.07	0.014
Methionine (%)	0.39ᵃ	0.38ᵃ	0.37ᵇ	0.36ᵃ	0.05	0.000
Methionine + cystine (%)	0.71ᶜ	0.69ᶜ	0.67ᵇ	0.65ᵃ	0.11	0.000
Threonine (%)	0.62ᵃ	0.61ᵃ	0.60ᵃ	0.59ᵃ	0.05	0.049
Tryptophan (%)	0.20ᵇ	0.19ᵃ	0.18ᵃ	0.18ᵇ	0.02	0.000

¹ME = metabolizable energy; SEM = standard error of the mean. Different superscripts within a row differ significantly (p < 0.05). T0–T3 denote fermented cassava peel (FCP) inclusion levels of 0%, 5%, 10%, and 15% in the diet.

### Production performance and feed efficiency

**Figure 1:**
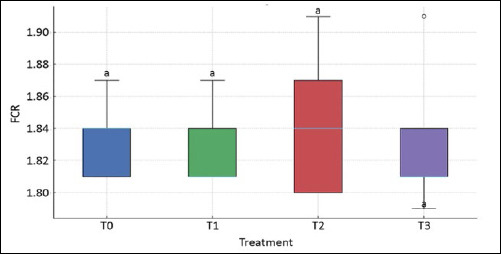
Feed conversion ratio of laying hens fed diets containing graded levels of fermented cassava peel. T0, T1, T2, and T3 represent dietary inclusion levels of fermented cassava peel at 0%, 5%, 10%, and 15%, respectively. Data are presented as mean ± SD. Means with no significant differences were observed among treatments (p > 0.05).

**Figure 2:**
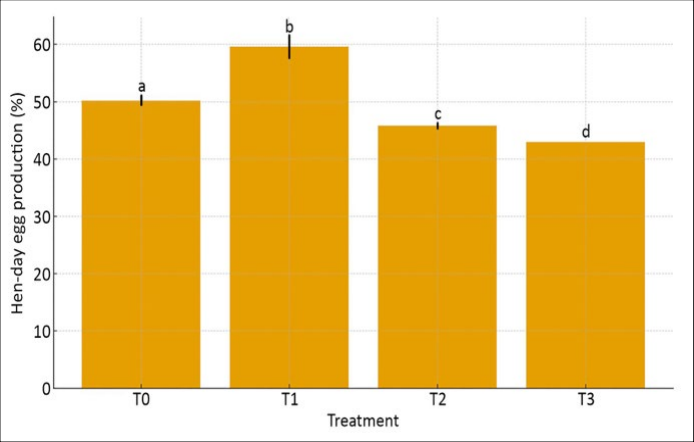
Hen-day egg production of laying hens fed diets containing graded levels of fermented cassava peel. T0, T1, T2, and T3 represent dietary inclusion levels of fermented cassava peel at 0%, 5%, 10%, and 15%, respectively. Data are presented as mean ± SD. Means with different superscript letters (a–d) above the bars differ significantly (p < 0.05).

As shown in Table 3 and Figures 1–2, *L. edodes*–FCP significantly affected egg production and hen-day production (HDP), whereas egg weight and FCR were not influenced (p > 0.05). Egg production was highest in hens fed 5% FCP (T1; 23.87 ± 0.79^b^ eggs/hen per period) and was significantly greater (p < 0.05) than in the control (T0; 20.06 ± 0.22^a^), 10% (T2; 18.26 ± 0.08^c^), and 15% FCP (T3; 17.21 ± 0.14^d^). Conversely, the lowest egg production was observed at 15% FCP, indicating that moderate inclusion improved output, whereas the highest level depressed it.

**Table 3:** Production performance of laying hens fed graded levels of *L. edodes*–fermented cassava peel diets.

Variable	T0	T1	T2	T3	SEM	p-value
Egg production (eggs/hen per period)	20.06ᵃ	23.87ᵇ	18.26ᶜ	17.21ᵈ	0.59	0.000
Egg weight (g)	54.61ᵃ	54.75ᵃ	54.27ᵃ	54.66ᵃ	0.23	0.916

¹FCP = fermented cassava peel; SEM = standard error of the mean. Different superscripts within a row differ significantly (p < 0.05). T0–T3 denote FCP inclusion levels of 0%, 5%, 10%, and 15% in the diet.

In contrast, egg weight did not differ among treatments (p > 0.05), with values ranging from 54.27 ± 1.35 g (T2) to 54.75 ± 0.85 g (T1), suggesting that FCP inclusion up to 15% did not alter individual egg size. Similarly, FCR remained statistically unchanged across diets (p > 0.05), indicating that the overall efficiency of converting feed into egg mass was maintained despite changes in egg number. However, HDP followed a pattern similar to egg production: hens receiving 5% FCP (T1) showed the highest HDP, which was significantly greater than T0, T2, and T3 (p < 0.05), while the lowest HDP occurred at 15% FCP.

### Yolk fatty acid composition

As shown in Table 4, inclusion of *L. edodes*–FCP in the diet selectively modified the yolk omega fatty acid profile, while total SFA and MUFA remained unchanged. The proportions of SFA and MUFA did not differ among treatments (p = 0.066 and p = 0.071), indicating that FCP up to 15% did not alter the overall saturation level of yolk lipids.

**Table 4:** Yolk fatty acids in egg yolk of laying hens fed diets with graded levels of FCP.

Variable	T0	T1	T2	T3	SEM	p-value
SFA (mg FA/100 g yolk)	7967.31ᵃ	7873.61ᵃ	8123.88ᵃ	8189.51ᵃ	48.13	0.066
MUFA (mg FA/100 g yolk)	1184.18ᵃ	1322.41ᵃ	1119.67ᵃ	1182.05ᵃ	28.84	0.071
Omega-3 (mg FA/100 g yolk)	75.20ᵃ	90.19ᵇ	80.11ᵃᵇ	87.15ᵇ	1.87	0.007
Omega-6 (mg FA/100 g yolk)	3241.26ᵃ	2931.54ᵇ	3406.35ᶜ	2608.40ᵈ	71.37	0.000
Omega-9 (mg FA/100 g yolk)	13518.13ᵃ	14118.92ᵇ	13487.48ᵃ	14132.29ᵇ	78.65	0.000

¹FCP = fermented cassava peel; SEM = standard error of the mean; FA = fatty acid; FAME = fatty acid methyl esters. Different superscripts within a row differ significantly (p < 0.05). T0–T3 denote FCP inclusion levels of 0%, 5%, 10%, and 15% in the diet.

In contrast, omega-3 and omega-9 fatty acids were significantly affected by FCP inclusion (p < 0.05). Yolk omega-3 content was higher in hens fed 5% and 15% FCP (T1 and T3) compared with the control (T0), whereas the 10% level (T2) was intermediate and did not differ from either group. A similar pattern was observed for omega-9, where T1 and T3 had higher proportions than T0 and T2 (p < 0.05).

Conversely, omega-6 fatty acids showed a marked and divergent response: T2 (10% FCP) had the highest omega-6 proportion, followed by T0, then T1, with the lowest value in T3 (p < 0.05; T2 > T0 > T1 > T3). Overall, these results suggest that FCP primarily modulated the balance among omega-3, omega-6, and omega-9 fatty acids in egg yolk, without significantly changing total SFA and MUFA levels.

### Correlations between production performance and yolk fatty acids

**Figure 3:**
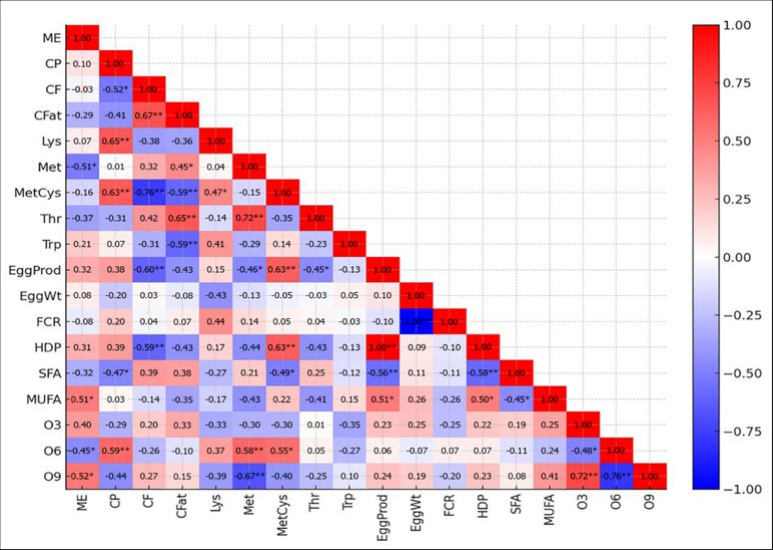
Pearson correlations between nutritional composition, production and efficiency with fatty acids in laying hens fed diets with graded levels of fermented cassava peel. Blue = negative, red = positive. Significance: * p < 0.05; ** p < 0.01; ME = metabolizable energy (kcal/kg); CP = crude protein (%); CF = crude fiber (%); CFat = crude fat (%); Lys = lysine (%); Met = methionine (%); MetCys = methionine + cystine (%); Thr = threonine (%); Trp = tryptophan (%); EggProd = egg production; EggWt = egg weight; SFA = saturated fatty acids; MUFA = monounsaturated fatty acids; O3 = omega-3 fatty acids; O6 = omega-6 fatty acids; O9 = omega-9 fatty acids.

As shown in Figure 3, Pearson correlation analysis revealed selective associations between yolk fatty acids and production performance. Yolk MUFA was positively correlated with egg production and HDP (r = 0.62, p = 0.018), whereas SFA was negatively correlated with both egg production and HDP (r = −0.54, p = 0.038). In contrast, egg weight and FCR were not significantly associated with any fatty acid class (p > 0.05).

### Economic outcomes

**Figure 4:**
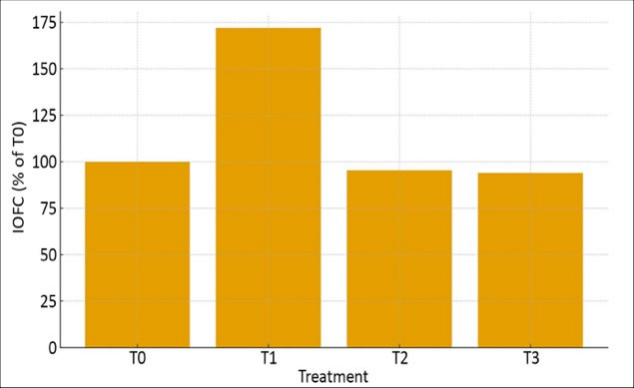
Income over feed cost of laying hens fed diets containing graded levels of fermented cassava peel. T0, T1, T2, and T3 represent dietary inclusion levels of fermented cassava peel at 0%, 5%, 10%, and 15%, respectively. Values are presented descriptively without replication.

Based on Figure 4, IOFC varied across FCP inclusion levels. Diet cost decreased progressively as *L. edodes*–FCP inclusion increased, reflecting partial substitution of concentrate with a lower-cost ingredient. Although feed cost per kilogram declined at 10% and 15% FCP, the greatest economic return was observed at 5% inclusion. Relative to the control (T0 = 100%), IOFC increased markedly at 5% FCP, reaching approximately 172% of the control. In contrast, IOFC at 10% and 15% inclusion levels declined below the control despite lower feed costs, indicating that reductions in egg production offset the economic benefit of further cost savings.

These results demonstrate that moderate inclusion (5%) provides the optimal economic balance between feed cost reduction and maintained laying performance, whereas higher inclusion levels reduce profitability due to diminished biological output.

## DISCUSSION

### Effects on laying performance

The present study indicates that *L. edodes*–FCP can be integrated into laying hen diets, achieving biological and economic optimality at approximately 5% inclusion. Egg production and HDP reached their highest levels at this dose, whereas egg weight and FCR were conserved across the tested range. This response pattern aligns with the behavior of fermented lignocellulosic co-products reported in poultry nutrition literature. Fermentation by edible fungi can open the plant cell wall, attenuate selected antinutritional factors, and generate small metabolites that improve palatability and gastric transit at moderate inclusion [6, 8]. At higher inclusion levels, the greater fiber bulk and potential changes in feed texture may have promoted earlier satiety despite diets being formulated to meet nutrient requirements. Increased dietary fiber can enhance gastrointestinal fill, slow digesta passage, and increase water-holding capacity in the gut, thereby limiting voluntary feed intake. This likely contributed to the observed decline in laying rate at 10–15% inclusion. However, FCR and egg weight remained unchanged across treatments, indicating that once nutrients were absorbed, the efficiency of converting consumed feed into egg mass was maintained. In this context, postabsorptive efficiency was inferred from stable FCR and egg mass output relative to intake, suggesting that metabolic utilization of absorbed nutrients was not impaired by higher fiber inclusion. The dose–response pattern observed in the present study aligns with earlier reports evaluating fermented cassava-based materials in poultry diets, in which moderate inclusion levels maintained production, whereas higher inclusion levels reduced laying performance. For example, Osei *et al*. [26] reported that increasing inclusion of FCP meal mixtures beyond moderate levels was associated with reduced production responses, which was attributed largely to increased dietary bulk and fiber-related constraints on intake. Similar trends have been noted in studies using fermented cassava by-products or other fibrous co-products, where the economic and biological optimum typically occurs at intermediate inclusion levels when nutrient requirements are met but physical limitations to intake are not exceeded [14, 27, 28].

### Changes in diet composition

Diet chemistry shifted in parallel with *L. edodes*–FCP level. CF and crude fat increased, whereas CP and lysine decreased, and metabolizable energy reached a modest peak near 5% inclusion. The observed decrease in CP and increase in crude fat with increasing *L. edodes*–FCP inclusion warrant careful interpretation. First, the reduction in CP primarily reflects the proportional replacement of protein-dense concentrate with a fiber-rich co-product rather than fermentation inefficiency per se. During solid-state fermentation, nitrogen losses may occur through microbial metabolism and ammonia volatilization, particularly when substrate nitrogen is limited; however, no signs of spoilage or abnormal odor indicative of excessive nitrogen loss were observed. The increase in crude fat may partly reflect a concentration effect due to dry matter reduction during fermentation, as degradation of structural carbohydrates can reduce total mass while retaining lipid fractions [29]. Additionally, fungal biomass contributes membrane lipids that may slightly elevate measured crude fat. From a dietary standpoint, these compositional shifts are physiologically coherent. Moderate inclusion may enhance lipid bioaccessibility and energy availability, whereas higher inclusion levels reduce the margin for essential amino acids due to partial replacement of concentrate with a fiber-rich ingredient [30]. When the supply of lysine and the sulfur amino acids becomes tighter, hepatic assembly of the very low-density lipoprotein particle that delivers yolk lipid can be constrained, which reduces the capacity to sustain an ovulatory rhythm even if egg size remains stable [31].

### Modulation of yolk fatty acid profile

Yolk fatty acid outcomes provide insight into how *L. edodes*–FCP influenced lipid deposition in laying hens. Although dietary crude fat increased with higher FCP inclusion, total yolk SFA and MUFA remained stable, suggesting regulatory homeostasis in overall lipid class distribution [32, 33]. In contrast, the omega series showed selective redistribution. Omega-6 closely reflected dietary supply because poultry depend largely on exogenous linoleic acid, whereas omega-3 shifts were modest in the absence of concentrated sources. Omega-9, largely oleic-acid related, appeared more buffered due to endogenous synthesis and redistribution among lipid pools [32, 33]. These changes occurred alongside increasing dietary fiber and crude fat, indicating that FCP inclusion influenced not only nutrient composition but also nutrient availability and partitioning. Fermentation-induced matrix modification, such as partial cell wall disruption, may enhance lipid release, micellization, and absorption efficiency even when absolute fatty acid supply is relatively unchanged [29, 30].

The correlation patterns suggest that yolk lipid distribution was associated with production responses. Higher yolk MUFA and lower SFA were aligned with improved laying rate, whereas omega-6 and omega-9 shifts tracked with differences in dietary energy and amino acid balance. These relationships likely reflect coordinated nutrient partitioning under requirement-balanced diets rather than direct causal mechanisms [10, 34]. Small variations in ME were associated with greater omega-9 representation, while sulfur amino acid supply showed associations with omega-6 deposition, supporting the concept that lipid routing in laying hens is sensitive to broader dietary context [32, 33]. Overall, the data indicate that FCP modulates yolk omega distribution through combined effects of dietary fat level, fiber inclusion, and fermentation-driven matrix changes, rather than through simple changes in total lipid intake.

### Economic implications

Consistent with these biological responses, the economic outcomes followed a similar pattern. IOFC was highest at five percent *L. edodes*–FCP, where moderate reductions in ration cost coincided with maintained or improved laying rate. In contrast, IOFC at ten to fifteen percent inclusion, the decline in egg production began to offset the additional feed cost savings, resulting in lower profitability relative to both the 5% treatment and, in some cases, the control diet. This response pattern aligns with previous findings showing that fermented agro-industrial by-products, including cassava peel and other lignocellulosic residues, tend to achieve maximum economic return at intermediate inclusion levels when production efficiency is maintained [27, 35, 36]. Although fermentation enhances their feeding value and allows partial replacement of conventional ingredients, excessive inclusion may impair intake or performance, thereby reducing overall profitability [35, 36].

From a practical standpoint, *L. edodes*–FCP serves as a readily available circular ingredient when used at around 5%. At this level, consumption remains steady, egg size is maintained, yolk lipid distribution shifts toward a profile with lower saturated and higher monounsaturated fractions that is favorable for sustained laying, and profitability improves. However, these recommendations should be interpreted with care. The composition and potential mycotoxin load of cassava peel can vary with cultivar, season, and processing, which may alter nutrient supply and palatability [37]. Relative prices of concentrates, energy sources, and *L. edodes*–FCP are volatile, therefore the IOFC advantage is sensitive to local markets. The response surface was characterized in a single strain, housing system, and climate, which narrows external validity. Formulation was requirement-based rather than digestibility-corrected, and the trial did not manipulate targeted lipid sources or essential amino acids to separate supply effects from matrix effects. If higher inclusion levels are required to meet cost or sustainability objectives, two safeguards are advisable. Reinforce lysine, methionine, and the methionine plus cystine pool to preserve hepatic lipoprotein assembly and ovulatory margin. Refine physical form and sensory attributes through particle size management, oiling, and flavor masking to limit satiety signals from fiber bulk [38].

### Limitations and future directions

Several limitations warrant consideration. First, the 12-week feeding period represents a defined production window but does not encompass a complete laying cycle; therefore, long-term effects on persistency, cumulative egg mass, and adaptation to dietary fiber remain uncertain. Second, apparent nutrient digestibility, gastrointestinal viscosity, and microbiota responses were not measured, limiting direct mechanistic interpretation of intake dynamics and nutrient utilization. Third, only a single *L. edodes* culture was evaluated, and strain-dependent variation in lignocellulolytic activity was not assessed. Additionally, the chemical composition of cassava peel may vary seasonally and by processing source, particularly in the fiber fraction and residual cyanogenic compounds, potentially affecting reproducibility across different field conditions. Future studies integrating digestibility trials, multi-strain fermentation comparisons, and extended production cycles would strengthen mechanistic resolution and external validity. In addition, absolute IOFC values and detailed ingredient price structures were not disclosed due to market variability and confidentiality constraints, and no formal sensitivity analysis or life-cycle environmental assessment was performed. Therefore, economic and sustainability interpretations should be considered within the scope of relative treatment comparisons rather than comprehensive bioeconomic or environmental modeling.

Future research should integrate intake monitoring with measurements of gastric emptying, digesta viscosity, apparent ileal digestibility, and zero Nitrogen retention (AMEn) to clarify how *L. edodes*–FCP influences gastrointestinal function and nutrient utilization, while enzymatic assays or stable isotope tracers may help resolve fatty acid partitioning and endogenous synthesis pathways underlying yolk lipid redistribution. A finer dose–response evaluation around three to seven percent inclusion could better define the biological and economic optimum. Although yolk lipid composition was assessed, broader egg quality parameters, including Haugh unit, albumen height, shell strength and thickness, yolk color, and sensory attributes, were not measured; therefore, conclusions regarding egg quality should be interpreted within the scope of lipid composition only. Gut microbiota and digesta characteristics were also not quantified, limiting the mechanistic interpretation of performance declines at higher inclusion levels. Finally, the 12-week feeding period represents a defined production phase rather than a complete laying cycle, and longer-term studies are needed to evaluate the persistence of lay, body condition, and sustainability under commercial conditions.

## CONCLUSION

This study demonstrated that dietary inclusion of *L. edodes*–FCP at 5% significantly improved laying performance in Hy-Line Brown hens, with egg production increasing to 23.87 eggs/hen per period and HDP reaching the highest level compared with the control and higher inclusion groups (p < 0.05). FCR and egg weight remained unaffected across treatments, while IOFC was maximized at approximately 172% of the control value at 5% inclusion. Higher levels (10% and 15%) reduced egg production and HDP, likely due to elevated dietary fiber content, despite progressive reductions in feed cost per kilogram. Yolk fatty acid profile was favorably modulated, with increased omega-3 and omega-9 proportions at 5% and 15% inclusion, while SFA and MUFA remained stable overall. Pearson correlations further revealed that higher yolk MUFA and lower SFA were positively associated with improved laying rate.

From a practical standpoint, *L. edodes*–FCP offers a viable, locally available agro-industrial byproduct for partial replacement of conventional ingredients in layer diets in tropical regions such as Indonesia. The 5% inclusion level optimizes both biological performance and economic return without compromising egg weight or feed efficiency, supporting sustainable, cost-effective egg production while enhancing yolk lipid quality by elevating omega-3 and omega-9 fatty acids. This approach contributes to circular bioeconomy strategies by valorizing abundant cassava peel waste.

The strength of the study lies in its well-controlled CRD with pen-level replication, comprehensive evaluation integrating production performance, yolk fatty acid profiling via GC-FID, and economic analysis (IOFC) under local market conditions, and confirmation of safety up to 15% inclusion with zero mortality.

Limitations include the relatively short 12-week experimental period, which does not cover a full laying cycle, lack of apparent digestibility or gut microbiota assessments to elucidate mechanisms behind intake depression at higher inclusions, use of a single *L. edodes* strain, and absence of detailed egg quality parameters beyond yolk lipids (e.g., shell strength, Haugh unit, sensory attributes).

Future research should focus on longer-term trials spanning complete laying cycles to evaluate persistency and cumulative egg mass, ileal digestibility and AMEn measurements, multi-strain fermentation comparisons, gut microbiota profiling, and broader evaluations of egg quality and sensory attributes. Finer dose–response studies around 3%–7% inclusion and economic sensitivity analyses under varying market prices would further refine practical recommendations.

In conclusion, *L. edodes*–FCP can be safely and profitably incorporated into laying hen diets at 5%, providing an effective strategy to reduce feed costs, improve production performance, and enhance yolk nutritional quality while promoting sustainable utilization of agro-industrial by-products. Gradual on-farm adoption with close monitoring of performance and economics is recommended.

## DATA AVAILABILITY

The supplementary data can be made available from the corresponding author upon reasonable request.

## AUTHORS’ CONTRIBUTIONS

JM, NN, DR, AMD, HH, MS, IWAD, WN, and MANJ: Conceived, designed, coordinated, and supervised the study. JM, NN, WN, and MANJ: Conducted the experiment and performed data collection and laboratory analyses. JM, AMD, NN, and MANJ: Statistical analysis, interpretation, and drafted the manuscript. All authors have read and approved the final version of the manuscript.
